# Association between MPO-463G > A polymorphism and cancer risk: evidence from 60 case-control studies

**DOI:** 10.1186/s12957-017-1183-7

**Published:** 2017-08-02

**Authors:** Wen-jun Yang, Ming-yue Wang, Fu-ze Pan, Chen Shi, Hong Cen

**Affiliations:** 1 Department of Gastroenterology, The Second Affiliated Hospital of Guilin Medical University, Gui Lin, 541100 China; 2 Department of Chemotherapy, Affiliated Tumor Hospital of Guangxi Medical University, Nanning, 530021 Guangxi China; 3 Department of Cardiology, The First Affiliated Hospital of Guangxi Medical University, Nanning, 530021 China; 4 Department of Gastroenterology, The First Affiliated Hospital of Guangxi Medical University, Nanning, 530021 China

**Keywords:** Gene polymorphism, Myeloperoxidase, Meta-analysis, Cancer

## Abstract

**Background:**

Though a number of studies have been conducted to explore the association between myeloperoxidase (MPO)-463G > A polymorphism and cancer risk, the results remain inconsistent. Therefore, we performed a meta-analysis to derive a more systematic estimation of this relationship.

**Method:**

Relevant studies were searched by PubMed, EMBASE, CNKI, Google Scholar, Ovid, and Cochrane library prior to December 2015. The strength of the association between MPO-463G > A polymorphism and cancer risk was estimated by odds ratios (OR) with 95% confidence interval (95%CI). Cumulative analysis was used to evaluate the stability of results through time.

**Results:**

The current analysis consisted of 16,858 cases and 21,756 controls from 60 studies. Pooled results showed that MPO-463G > A polymorphism were associated with the overall decreased cancer susceptibility in all the genetic models included in this study (additive model: OR = 0.84, 95%CI = 0.76–0.94; allele genetic model: OR = 0.90, 95%CI = 0.840–0.954; recessive genetic model: OR = 0.89, 95%CI = 0.83–0.95). However, in the stratified analysis of cancer type, the significant results were only found in lung cancer (dominant model: OR = 0.93, 95%CI = 0.87–0.99) and digestive system cancer groups (dominant model: OR = 0.67 0.53–0.84; allele frequency model = 0.71, 95%CI = 0.57–0.87), but not in the blood system cancer or breast cancer group. When we further stratified the digestive system cancer group into digestive tract and digestive gland cancer groups, results showed a significant association between allele A of MPO-463G > A and digestive gland cancer in all the genetic models (allele frequency model: OR = 0.63, 95%CI = 0.40–0.99; additive model: OR = 0.41, 95%CI = 0.23–0.73; recessive model: OR = 0.51, 95%CI = 0.29–0.89; dominant model: OR = 0.58, 95%CI = 0.35–0.96), digestive tract cancers in allele frequency model (OR = 0.75, 95%CI = 0.59–0.95), and dominant model (OR = 0.72, 95%CI = 0.56–0.92). When stratified by ethnicity, results demonstrated that the genotype A might be a protect factor for both Caucasians and Asians. In group analysis according to source of controls, significant results were found in population from hospital in all the genetic models. In cumulative analysis, result of allele contrast showed a declining trend and increasingly narrower 95% overall, while the inclination toward non-significant association with lung cancer risk.

**Conclusions:**

This meta-analysis suggested that MPO-463G > A polymorphism was associated with the overall reduced cancer susceptibility significantly. It might be a more reliable predictor of digestive system cancer instead of lung cancer, blood system cancer, and breast cancer. In cumulative analysis, the stable trend indicated that evidence was sufficient to show the association between MPO-463G > A polymorphism and cancer risk.

## Background

Cancer is a multifactorial disease resulted from complex interactions between genetic and environmental and its worldwide burden is increasing [1]. Reactive oxidative stress (ROS) is showed to play a significant role in immunological defense, intracellular signaling, and intercellular communication [2]. An increased ROS is considered to be mutagenic and carcinogenic, and it may result in cell damage and alterations of the cell proliferation and apoptosis mechanism, thus contributing to cancer development [3].

Myeloperoxidase (MPO) is an endogenous metabolic/oxidative lysosomal enzyme secreted by reactive neutrophils and monocytes. MPO plays an important role in carcinogenesis through activating procarcinogens to genotoxic intermediates and potentiation of xenobiotic carcinogenicity [4]. The MPO-463G > A polymorphism is located in the promoter region of MPO gene and is most extensively studied. This polymorphism may influence the MPO transcription level through removing a binding region for transcription factor Sp1, which can decrease the metabolic activation of carcinogenic compounds. Studies suggested that the MPO A allele carriers were associated with lower mRNA expression and transcription activity than the 463 G common allele [5–7]. Abundant studies were conducted to investigate the role of this polymorphism in cancer development, including lung cancer, breast cancer, ovary cancer, gastric carcinoma and others, but results were inconsistent.

For instance, the association between MPO-463G > A polymorphism and lung cancer risk has attracted the most attention since the first meta-analysis performed in 2002 and suggested a slight protective effect of the MPO 463 A variant [8]. But research results in the following years varied a lot. Whether MPO-463G > A polymorphism could be a good predictor of cancer remained controversial. Up to 2010, a meta-analysis gave a systematic review into the association between MPO-463G > A polymorphism and cancer risk, including breast cancer, leukemia, and lung cancer [9]. This analysis did not report a significant association between these cancers and MPO-463G > A polymorphism, but suggested a moderately protective effect on cancer risk in Europeans. After that, many new studies attempted to further describe this association emerged, but came up with different results [10, 11].

In consideration of the recently published studies and the absence of a new systematic and comprehensive evaluation for the association between MPO-463G > A polymorphism and cancer risk, we conducted this meta-analysis to shed a light on this relationship.

## Methods

### Search strategy

Eligible literatures published before December 2015 were identified by searching PubMed, Cochrane Clinical Trials Database, Medline, EMBASE Google Scholar, and the Ovid Library with the following keywords: “Myeloperoxidase”, “MPO”, “polymorphism,” or “variant” without any restriction on language. The scope of computerized literature search was expanded according to the reference lists of retrieved articles. The relevant original articles were also retrieved manually.

### Inclusion and exclusion criteria

Any observational studies met the following criteria were included: (a) a study related to the MPO-463G > A polymorphism and cancer risk, (b) a case-control study, (c) the data of genotype frequency was available. Major reasons for exclusion of studies were as follows: (a) the use of only case-group data, (b) the duplication or overlap of previous publication, (c) the Newcastle-Ottawa Scale (NOS) score is less than 5 stars, and (d) familial type of cancer.

### Data extraction

After removing duplicate studies, two investigators extracted the data individually from the reserved studies, and a third investigator was involved when discrepancies were raised. From each study, the following information was extracted: first author, journal, year of publication, country, ethnicity (Caucasian, Asian, and others), cancer type, source of controls (hospital-based studies, population-based studies), identification of cancer cases, genotyping methods, and the number of cases and controls for MPO-463G > A polymorphism.

### Quality assessment

The Newcastle-Ottawa Scale (NOS) is a star rating system used to assess the study quality. The full score is defined as 9 stars, and 0 to 4 stars are usually considered to be a poor methodological quality while 5 to 9 stars are considered to be high quality. Any disagreements on the NOS score of these studies were solved by discussion and only studies with high quality were included.

### Statistical analysis

The frequencies of the alleles and distributions of the genotypes of MPO-463G > A polymorphism in control groups were tested for Hardy-Weinberg equilibrium (HWE) using *X* ^2^ test. Odds ratios (OR) and 95% confidence intervals (95%CI) were used to assess the strength of associations between MPO-463G > A polymorphism and cancer risk. The combined ORs were calculated for dominant model (AA/GA versus GG), recessive model (AA versus GA/GG), additive model (AA versus GG), and allele frequency model (A versus G) to assume the effects of the variant A allele, respectively. Stratified analyses were also conducted by cancer types, ethnicity, HWE, and source of controls.

The heterogeneity between studies was tested using the *Q*-statistic. *I* ^2^ metrics was used to determine the impact of heterogeneity and it was considered moderately when *I* ^2^ < 50%, and a fixed-effects model was utilized. Heterogeneity was considered statistically significant when *I* ^2^ > 50% and a random-effects model was employed to calculate the pooled ORs [12]. Sensitivity analysis was carried out to evaluate the influence of individual study by excluding each study at a time. Publication bias was investigated with funnel plots and Egger’s tests qualitatively. In addition, in order to evaluate the trend in OR over time, cumulative meta-analysis was performed. All statistical analyses were performed using STATA 11.2 statistical software.

## Results

### Literature search results and studies characteristics

**Fig. 1:**
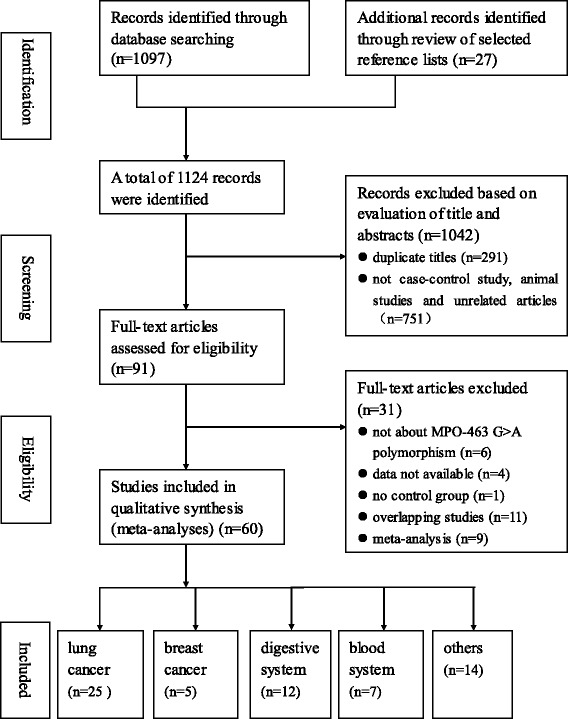
Flow chart illustrating the identification, screening, eligibility, and inclusion of studies on MPO-463G > A polymorphism and cancer risk

After a comprehensive literature search, 1124 independent studies were preliminarily found. After excluding the uncorrelated and duplicate articles, 91 articles were included. Through reading the full texts, we further excluded 31 articles. Among them, 6 were not about MPO-463G > A polymorphisms, 11 had overlapping data, 4 did not report available data, 9 were meta-analyses, and 1 was not a case-control study. Therefore, the current meta-analysis included data from 60 articles that consisted of 38,614 subjects (16,858 cases and 21,756 controls). Figure 1 provides a summary of the selection process.

Table 1 shows a list of details of studies included in the current meta-analysis. The included articles comprised 25 lung cancer studies [8, 13–36], 5 breast cancer studies [37–41], 12 digestive system studies (gastrointestinal cancer: 3 esophagus studies, 3 stomach studies, and 2 colorectum studies; cancer of digestive organs: 3 liver studies, 1 pancreas studies) [42–53], 7 blood system studies (3 acute leukemia studies, 3 lymphoma studies, 1 multiple myeloma, and chronic granulocytic leukemia studies) [54–60] and 14 other studies ( 3 head and neck cancer studies, 3 gynecological tract cancer studies, 3 urological cancer studies, 2 musculoskeletal cancer studies, and 3 mix cancer studies) [14, 34, 61–71]. Cancers referred in the mix cancer studies included lung cancer, laryngeal cancer, and pharyngeal cancer [14]; lung cancer, prostate cancer [34]; multiple myeloma, and chronic granulocytic leukemia [58]. If the ethnicity was clearly described in the original studies, the studies would be categorized to the corresponding ethnicity group. If the original studies have not given a clear indication of ethnicity, the studies were categorized according to research region. If it has been specified in the original studies that the objects included were from different ethnicities, then studies should be categorized to the mix ethnicity group. Of the 60 studies, 24 were Asian background, 32 were Caucasian background, and 4 were mixed population background. Besides, the detailed description of hospital controls was addressed in Table 4.

**Table 1:** Overview of studies included in the current meta-analysis

Author	Year	Country	Ethics	Cancer	Disease confirmed	Resource	Case	Control	Genotyping	NOS score
London	1997	America	mix Ethics	Lung cancer	NA	PB	339	703	RT-PCR	7
Marchand	2000	America	mix Ethics	Lung cancer	Histological	PB	323	437	PCR-RFLP	8
Misra	2001	Finland	Caucasian	Lung cancer	Histological	PB	315	311	PCR-RFLP	8
Xu	2002	America	Caucasian	Lung cancer	Histological	HB	989	1128	PCR-RFLP	7
Lv	2002	China	Asian	Lung cancer	Histological	PB	314	320	PCR	8
Kantarct	2002	America	Caucasian	Lung cancer	NA	HB	307	307	NA	5
Feyler	2002	France	Caucasian	Lung cancer	Histological	HB	150	172	capillary PCR	7
Dally	2002	Germany	Caucasian	Lung cancer	NA	HB	625	340	PCR-RFLP.	6
Chevriera	2003	France	Caucasian	Lung cancer	Histological	HB	243	245	PCR-RFLP	7
Skuladottira	2004	Denmark	Caucasian	Lung cancer	NA	PB	122	396	NA	6
Chana	2004	China	Asian	Lung cancer	NA	HB	75	162	PCR-RFLP	6
Harms	2004	America	Caucasian	Lung cancer	Pathological	PB	110	119	MALDI-TOF	8
Wu	2004	China	Asian	Lung cancer	Histological	PB	98	112	PCR	8
Schabath	2005	America	Caucasian	Lung cancer	Histological	HB	837	618	PCR-RFLP	7
Park	2006	Korea	Asian	Lung cancer	Histological	HB	432	432	PCR-RFLP	7
Larsen	2006	Australia	Caucasian	Lung cancer	Cytological or histological	HB	627	624	PCR	7
Yanga	2007	Korean	Asian	Lung cancer	Histological	HB	318	353	PCR-RFLP	7
Zienolddiny	2008	Norway	Caucasian	Lung cancer	Pathologists	PB	258	297	NA	7
Yoon	2008	Korea	Asian	Lung cancer	Histological	HB	213	213	Taqman probe	7
Rotunno	2009	America	Caucasian	Lung cancer	Histology	PB	185	2011	NA	7
Klinchid	2009	Thailand	Asian	Lung cancer	Histological	HB	88	81	PCR-RFLP	7
Kiyohara	2014	Japan	Asian	Lung cancer	Histological	HB	462	379	TaqMan	7
Bag	2014	India	Asian	Lung cancer	Tissue diagnosis	HB	26	37	PCR-RFLP	7
Cascorbi	2000	Germany	Caucasian	Mix	NA	PB	696	270	PCR-RFLP	7
Arslan	2011	Turkey	Caucasian	Mix	Histological	HB	220	418	PCR-RFLP	7
Lin	2004	Taiwan	Asian	Breast cancer	Medical charts and pathology	PB	99	366	PCR-RFLP	8
Ahn	2004	Canada	Caucasian	Breast cancer	NA	PB	1011	1067	PCR-RFLP.	7
Yang	2007	America	Caucasian	Breast cancer	NA	PB	406	392	PCR-RFLP	7
Li	2009	America	Caucasian	Breast cancer	NA	NA	477	462	PCR-RFLP.	5
Tsai	2012	Taiwan	Asian	Breast cancer	NA	NA	260	224	PCR-RFLP	5
Zhang	2007	China	Asian	Acute leukemia	French-American-British criteria	HB	135	187	Taqman	7
Krajinovic	2002	Canada	Caucasian	ALL	Hematology-oncology	HB	169	337	PCR-RFLP	7
Silveira	2010	Brazil	mix Ethics	ALL	Immunophenotyping	NA	124	300	PCR-RFLP	6
Matsuo	2001	Japan	Asian	Lymphoma	Histological	HB	372	241	PCR-RFLP	7
Saygilii	2009	Turkey	Caucasian	Mix	NA	HB	62	40	TaqMan	6
Wang	2006	America	Caucasian	NHL	Histopathology	PB	1082	905	NA	7
Farawela	2012	Egypt	Asian	NHL	Biopsy	HB	100	100	RT-PCR	7
Funke	2009	Germany	Caucasian	Colorectal cancer	NA	PB	627	603	PCR	7
Li	2011	China	Asian	Colorectal cancer	Histological	HB	325	345	PCR-RFLP	7
Matsuo	2001	Japan	Asian	Esophageal cancer	NA	HB	91	241	PCR-RFLP	6
Li	2008	China	Asian	Esophageal cancer	Pathologists	PB	126	169	PCR-RFLP	8
Li	2011	China	Asian	Esophageal cancer	Histopathologic	HB	94	280	NA	6
Zhu	2006	China	Asian	Gastric cancer	Biopsy or surgical specimens	NA	127	139	PCR-RFLP	6
Wang	2011	China	Asian	Gastric cancer	Pathological	HB	62	61	ASP-PCR	7
Jang	2012	China	Asian	Gastric cancer	Pathological	HB	117	105	PCR-RFLP	7
Pakakasama	2003	America	Caucasian	Hepatoblastoma	PCR-SSCP	PB	48	180	Pyrosequencing	7
Nahon	2011	France	Caucasian	Hepatocellular cancer	Barcelona criteria	HB	84	121	NA	6
Carmo	2012	Brazil	Caucasian	Hepatocellular cancer	AASLD guidelines	HB	32	252	PCR-amplified	7
Mustea	2007	Germany	Caucasian	Cervical cancer	Histological	HB	149	126	PCR-RFLP	7
Olson	2004	America	Caucasian	Ovarian cancer	NA	PB	122	179	TaqMan	7
CastilloTong	2014	Austria	Caucasian	Ovarian cancer	Histopathological	NA	305	299	TaqMan probes	6
Hung	2004	Italy	Caucasian	Bladder cancer	Histological	HB	201	214	PCR-RFLP	7
Hsieh	2010	Taiwan	Asian	Leiomyoma	Pathological	HB	158	156	PCR-RFLP	7
Guo	2010	China	Asian	Nasopharyngeal carcinoma	Biopsy	HB	358	629	PCR-RFLP	7
Wu	2010	Taiwan	Asian	Oral cavity	Pathological	HB	122	122	PCR	7
Oliveira	2007	Brazil	mix Ethics	Osteosarcoma	NA	HB	78	157	PCR-RFLP	6
Price	2008	Canada	Caucasian	Pancreatic cancer	Histological	HB	122	331	PCR	7
Buch	2008	America	Caucasian	Squamous cell carcinoma	Biopsy-verified	PB	193	414	PCR-RFLP	8
Choi	2008	America	Caucasian	Prostate cancer	Pathology	HB	493	1332	NA	6
Tefik	2013	Turkey	Caucasian	Prostate cancer	Histological	HB	155	195	PCR	7

*NA* not available, *ALL* acute lymphoblastic leukemia, *NAL* non-Hodgkin lymphoma, mix: 2 or more cancer type, *PB* population base, *HB* hospital base, *PCR-RFLP* polymerase chain reaction restriction fragment length polymorphism, *RT-PCR* real-time polymerase chain reaction

### Quantitative synthesis

**Fig. 2:**
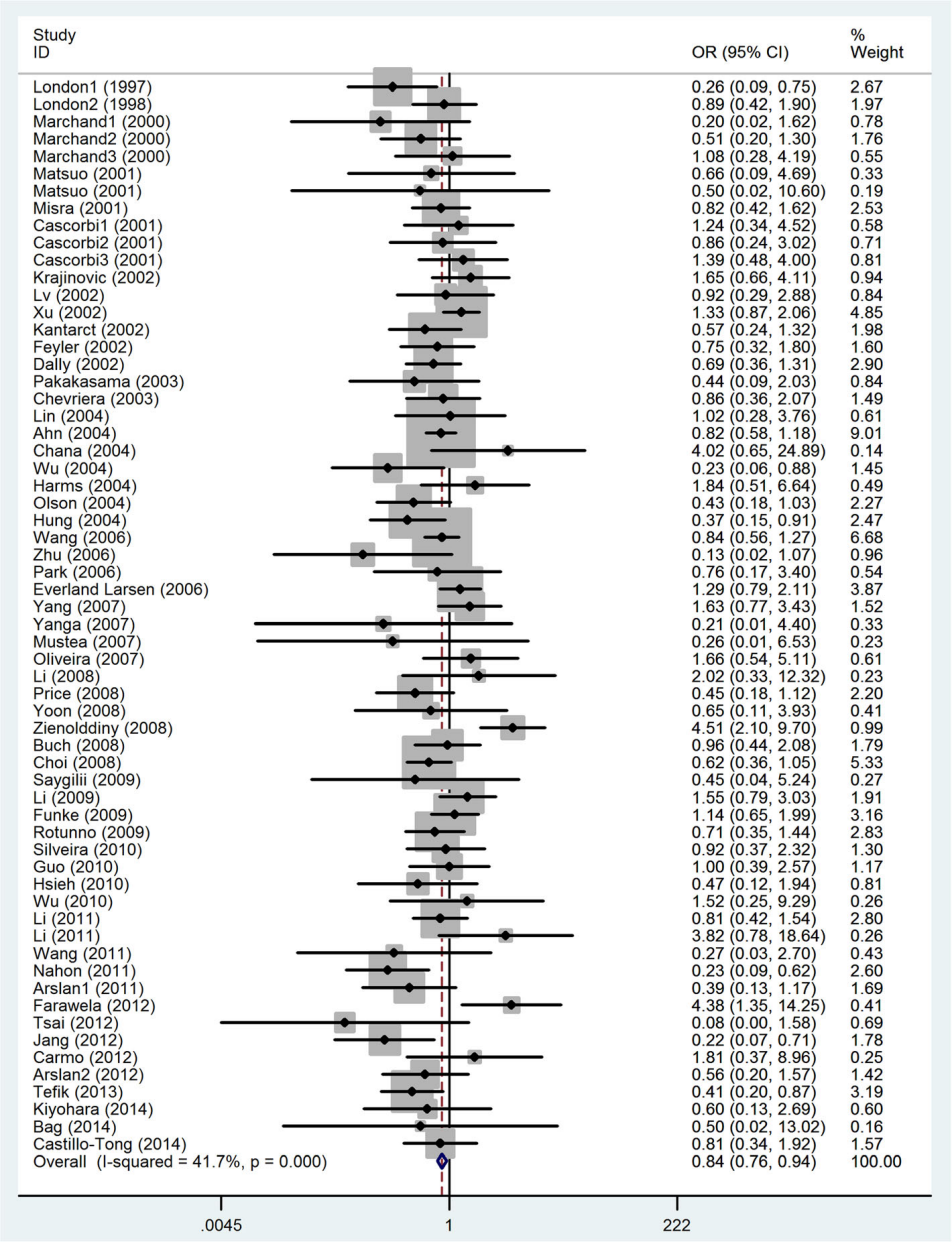
Forest plots of meta-analysis of MPO-463G > A polymorphism in association with cancer risk (AA vs. GG)

In total, significant association between MPO-463G > A polymorphism and cancer risk was observed under all the selected models when the eligible results were pooled together (Fig. 2). Results promoted that the mutant allele A and genotypes of AA as well as AA + GA might played protective roles in the development of cancer (AA versus GG: OR = 0.84, 95%CI = 0.76–0.94; A versus G: OR = 0.90, 95%CI = 0.84–0.95; AA versus AG/GG: OR = 0.87, 95%CI = 0.78–0.97; AA/AG versus GG: OR = 0.89, 95%CI = 0.83–0.95). The pooled results are summarized in Table 2.

**Table 2:** Stratified analyses of the MPO-463G > A polymorphism on cancer risk by cancer type

Study groups	A vs. G		AA vs. GG		AA vs. AG + GG		AA + AG vs. GG	
	OR (95%CI)	*I* ^2^	OR (95%CI)	*I* ^2^	OR (95%CI)	*I* ^2^	OR (95%CI)	*I* ^2^
Total	0.90(0.84–0.95)	0.571	0.84(0.76–0.94)	0.417	0.87(0.78–0.97)	0.366	0.89(0.83–0.95)	0.520
Lung	0.92(0.83–1.01)	0.525	0.93(0.78–1.10)	0.481	0.95(0.80–1.12)	0.488	0.93(0.87–0.99)	0.420
Breast	0.98(0.83–1.16)	0.545	0.99(0.75–1.30)	0.441	1.00(0.76–1.32)	0.342	0.96(0.85–1.08)	0.418
Digestive	0.71(0.57–0.87)	0.694	0.63(0.37–1.05)	0.529	0.77(0.57–1.02)	0.415	0.67(0.53–0.84)	0.619
Dig tract	0.75(0.59–0.95)	0.668	0.74(0.39–1.43)	0.523	0.92(0.65–1.29)	0.449	0.72(0.56–0.92)	0.577
Dig gland	0.63(0.40–0.98)	0.700	0.41(0.23–0.72)	0.355	0.51(0.29–0.89)	0.000	0.58(0.35–0.96)	0.649
Blood	1.12(0.96–1.25)	0.431	0.96(0.63–1.58)	0.501	1.03(0.85–1.40)	0.623	1.03(0.85–1.40)	0.623

### Stratified by cancer type

According to the cancer type, when the minimum number of studies is greater than or equal to 5, we combined the studies and conducted stratified analysis. Considering that gastrointestinal and digestive organs all belong to digestive system and might be affected by similar factors such as dietary factors, we grouped them together as digestive system cancer group. In the digestive system cancer group, significant association was seen in the allele frequency model (OR = 0.71, 95%CI = 0.57–0.88) and the dominant model (OR = 0.67, 95%CI = 0.53–0.84). In order to further discuss the cancer risk of MPO-463G > A polymorphism, we divided digestive system cancer into digestive tract and digestive gland cancer. The results showed that allele A of MPO-463G > A was significant with a decreased risk of digestive gland cancer in all the genetic models (A versus G: OR = 0.63, 95%CI = 0.40–0.99; AA versus GG: OR = 0.41, 95%CI = 0.23–0.73; AA versus AG/GG: OR = 0.51, 95%CI = 0.29–0.89; AA/AG versus GG: OR = 0.58, 95%CI = 0.35–0.96) and digestive tract cancers (A versus G: OR = 0.75, 95%CI = 0.59-0.95; AA/AG versus GG:OR = 0.72, 95%CI = 0.56–0.92). Besides, in the lung cancer group, statistically significant finding was only observed in dominant model (OR = 0.93, 95%CI = 0.87–0.99). While, among studies of breast cancer and blood system cancer, no significant association was found in any genetic model.

### Stratified by ethnicity

In terms of ethnicity, we divided the ethnicity into three groups: Caucasian group, Asian group, and mix ethnicity group. The current result demonstrated that the genotype A may be a protect factor for both Caucasians and Asians, but not for mix ethnicity group. The different result from previous meta-analyses is seen in Table 3.

**Table 3:** Stratified analyses of the MPO-463G > A polymorphism on cancer risk by ethnicity and source of control group

Variables	A vs. G		AA vs. GG		AA vs. AG + GG		AA + AG vs. GG	
	OR (95%CI)	*I* ^2^	OR (95%CI)	*I* ^2^	OR (95%CI)	*I* ^2^	OR (95%CI)	*I* ^2^
Total	0.90(0.854-1.069)	0.571	0.84(0.76-0.94)	0.417	0.89(0.83-0.95)	0.520	0.87(0.78-0.97)	0.366
Ethnicity								
Caucasian	0.90(0.84–0.97)	0.576	0.85(0.76–0.96)	0.506	0.91(0.84–0.98)	0.533	0.87(0.78–0.98)	0.484
Asian	0.86(0.75–0.98)	0.597	0.75(0.57–0.98)	0.353	0.84(0.73–0.96)	0.516	0.80(0.61–1.05)	0.242
Other	1.05(0.86–1.27)	0.000	1.03(0.64–1.67)	0.000	1.08(0.85–1.37)	0.000	0.98(0.61–1.57)	0.000
Lung cancer								
Caucasian	0.956(0.84–0.97)	0.524	1.082(0.888–1.319)	0.573	1.050(0.749–1.472)	0.593	0.950(0.878–1.029)	0.420
Asian	0.863(0.691–1.078)	0.551	0.641(0.370–1.110)	0.013	0.709(0.400–1.256)	0.000	0.861(0.740–1.002)	0.495
Other	0.861(0.715–1.037)	0.176	0.567(0.363–0.886)	0.000	0.558(0.360–0.867)	0.000	0.926(0.758–1.132)	0.467
Digestive Cancer								
Caucasian	0.704(0.474–1.045)	0.796	0.609(0.291–1.274)	0.620	0.751(0.514–1.100)	0.428	0.658(0.423–1.024)	0.756
Asian	0.706(0.537–0.928)	0.613	0.640(0.271–1.512)	0.533	0.785(0.507–1.215)	0.491	0.669(0.512–0.875)	0.474
Resource								
Population	0.91(0.83–0.99)	0.541	0.90(0.77–1.05)	0.453	0.91(0.81–1.01)	0.518	0.91(0.78–1.06)	0.461
Hospital	0.89(0.81–0.98)	0.603	0.79(0.68–0.93)	0.400	0.88(0.80–0.98)	0.543	0.83(0.71–0.96)	0.301
Others	0.86(0.69–1.08)	0.625	0.87(0.57–1.33)	0.525	0.86(0.68–1.08)	0.503	0.90(0.59–1.36)	0.111
HWE								
Yes	0.89(0.83–0.96)	0.598	0.88(0.77–0.96)	0.454	0.88(0.81–0.95)	0.507	0.92(0.81–1.04)	0.373
No	0.91(0.81–1.02)	0.434	0.75(0.61–0.93)	0.167	0.93(0.79–1.10)	0.607	0.74(0.60–0.91)	0.297

When further stratified, the digestive system cancer subgroup by ethnicity, no significant association between MPO-463G > A polymorphism and Caucasians was found in any genetic model. Conversely, for the lung cancer subgroup, there was no significant association between MPO-463G > A polymorphism and Asians (Table 3).

### Stratified by HWE

Not all the studies included in this current analysis conformed to HWE. When stratified, in the HWE balanced group, the MPO-463G > A polymorphism was significantly associated with cancer risk in all the genetic models, while in the HWE un-balanced group, this association was only found in the recessive model and additive model. Results were represented in Table 2.

### Stratified by source of control group

When it was stratified according to the source of control group, a statistically significant finding was found among hospital controls (A versus G: OR = 0.892, 95%CI = 0.811–0.981; AA versus GG: OR = 0.79, 95%CI = 0.68–0.93; AA/AG versus GG : OR = 0.88, 95%CI = 0.80–0.97; AA versus AG/GG: OR = 0.83, 95%CI = 0.71–0.96), but no statistically significant evidence was found in controls based on population under most of the selected gene models except allele frequency model (OR = 0.91, 95%CI = 0.83–0.97) (Table 4).

**Table 4:** The hospital control’s details

Author	Year	details
Matsuo	2001	Outpatients without any history of cancer
Matsuo	2001	Non-cancer controls
Xu	2002	Friends or spouses of patients (with either lung cancer or other cardiothoracic problems), with no matching characteristics
Krajinovic	2002	Selected from a large institutional DNA bank. Care was taken to match the patient population by selecting controls of French-Canadian origin served by Sainte-Justine Hospital
Kantarct	2002	Without diagnosis of lung cancer
Feyler	2002	Frequency matched on age, sex, and hospital, consisted of all consecutive Caucasian patients without previous or current malignant diseases
Dally	2002	Had no previous or present history of malignant diseases: the main diagnoses included alveolitis, bronchitis,pneumonia, fibrosis,sarcoidosis, COPD and emphysema
Chevriera	2001	All subjects hospitalized for different disorders except cancer
Chana	2004	Had no history of pulmonary diseases, and were receiving health evaluation for other reasons and matched for sex and age with the lung cancer patients
Hung	2004	Patients admitted to the same hospitals during the same period of time, with urological non-neoplastic diseases, including hydronephrosis, urolithiasis, malformative urological diseases, prostatic adenoma, and hypertrophia, urological traumas, orchiepididymitis, hydrocele and unspecified urinary symptoms
Schabath	2005	Healthy controls frequency matched to the cases on age (±5 years), gender,ethnicity, and smoking status (current, former, and never)
Park	2006	Healthy volunteers
Larsen	2006	Controls consisted of patients with chronic obstructive pulmonary disease (COPD) but without lung cancer (*n* ¼ 380), treated at the same hospital from 1998 to 2003, or healthy smokers attending a smoking cessation clinic held at the hospital from 2000 to 2003
Zhang	2007	No known malignant diseases
Yanga	2007	Healty individuals without lung cancer or any other cancer
Oliveira	2007	Individuals admitted in the Pediatric department of the Federal University of Sao Paulo, Brazil without osteosarcoma
Mustea	2007	The control group consisted of similarly aged women with no history of cancer and all of them were treated for benign gynecological diseases. None of them had previously undergone a hysterectomy
Yoon	2008	Healthy control
Price	2008	Healthy controls were frequency matched for age and sex. The controls were healthy nonblood-related family members (usually spouses) and friends of other cancer/surgical patients and were used as a shared set of controls for aerodigestive cancers
Choi	2008	Free of both prostate cancer and lung cancer
Saygilii	2009	Healthy volunteers and No one in the control group had a smoking history or chronic use of any drugs
Klinchid	2009	Healthy volunteers and diabetic patients
Wu	2010	With the same habits and without a present or previous history of any cancer
Hsieh	2010	Non-leiomyoma
Guo	2010	Spouse or geographically matched residents who were EBV/IgA/VCA positive (IgA+) or EBV/IgA/ VCA negative (IgA-) and NPC free at the time of study enrollment
Nahon	2011	HCV-induced cirrhosis
Wang	2011	Non-cancer controls
Li	2011	Healthy had no current or previous diagnosis of cancer and genetic disease
Li	2011	Diagnosed as normal by histopathology of ophageal squamous epithelial cells
Arslan	2011	Healthy individuals without any history of cancer
Jang	2012	Individuals without gastic cancer
Carmo	2012	They had persistent anti-HCV antibodies and were HCVRNA positive. Presence of hepatitis A, hepatitis B, and immunodeficiency virus (HIV) antibodies were considered as exclusion criteria
Tefik	2013	Normal DRE and serum PSA levels of <4 ng/mL
Bag	2014	Normal healthy individuals with no history of cancer
Kiyohara	2014	Without a clinical history of any type of cancer past or present, ischemic heart disease or chronic respiratory diseases

### Cumulative meta-analysis

**Fig. 3:**
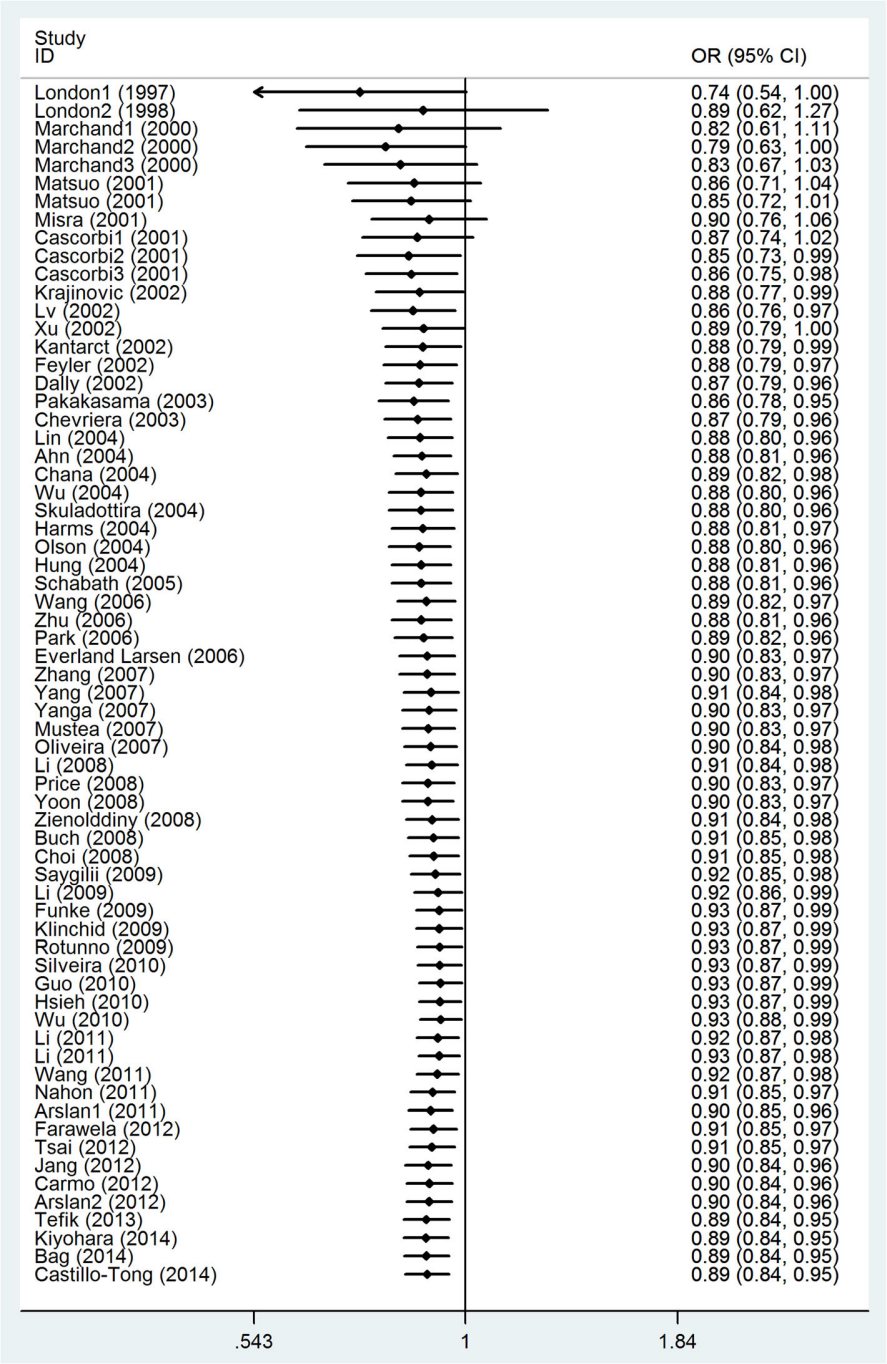
Cumulative meta-analysis of MPO-463G > A polymorphism in association with cancers by published year (A vs. G)

We performed cumulative meta-analyses for both overall cancer risk and lung cancer risk. For the former, as was shown in Fig. 3, the cumulative result of allele contrast showed a declining trend in the estimated protective effect in the vicinity of 0.890 during 2001 and 2014. And the relative change in the random-effects ORs was lower than 1.0 since 2001. Further, the increasingly narrower 95%CIs suggested the precision of the estimates was gradually improved by continually adding more samples. In the same gene model, the inclination toward a non-significant association with overall lung cancer risk except for adding data provided by Heike in 2002 and Isabelle in 2003.

### Sensitivity analyses and publication bias

Each included study was deleted one by one to reflect the influence of the individual data on the pooled ORs, and the corresponding results were not significantly changed. Moreover, there was no influence of publication bias in our study by using Begg’s test or Egger’s tests.

## Discussion

The current analysis provided the most comprehensive investigation of MPO-463G > A polymorphism and cancer risk. Sixty studies consisted of 16,858 cases and 21,756 controls gave greater information to explore the association; however, the previous meta-analysis published in 2010 included only 43 studies with 14,171 cancer cases and 17,319 controls. The methodology used for this meta-analysis and the statistical evaluation of the results were well-developed. This meta-analysis demonstrated a new subgroup (digestive system cancer group) to discussed the association between MPO-463G > A polymorphism and cancer risk in depth. In addition, we further stratified the digestive system cancer and lung cancer group by ethnicity and found some interesting results that the protect effect of MPO-463G > A polymorphism was only found in Caucasians in lung cancer population and Asians in digestive system cancer population. Furthermore, the cumulative meta-analysis which had not been conducted in previous relevant meta-analysis provided more powerful evidence that the results were reliable. Moreover, NOS was used to evaluate the quality of the studies, and results suggested that all the studies included in the current meta-analysis were high quality.

Contrary to the earlier meta-analysis which indicated that no significant association was found in any genetic model, the current meta-analysis showed evidence that allele A was associated with a reduced cancer risk compared with G allele. Besides, in view of the cumulative analysis results, we intended to draw a conclusion that MPO-463G > A polymorphism had a protective significance of cancer risk. But when the studies were stratified by cancer type, ethnicity, HWE, and control resource, results differed.

Among all the cancers studied in our meta-analysis, lung cancer was mostly discussed. We were interested to find that the results of three related meta-analyses published in 2013, were not identical with each other. Zhou YY et al. found MPO-463G > A polymorphism was significantly associated with decreased risk of lung cancer risk in Asians under additive model and recessive model [72]. Zhou C et al. suggested that in allele frequency and dominant models, when stratified by ethnicity, evidence showed a protect effect in Caucasians, but not in Asians [73]. While, Yang et al. indicated there was no significant association of both overall and stratified analyses according to ethnicity, source of controls and smoking status [74]. What is more, another two meta-analyses published in 2014 by Li and Huang et al. [75, 76], respectively, also got different conclusions. The varied results may have relation with the different search strategies, selection criteria, quality of the original studies and so on. Considering the confusing outcome of the lung cancer, we further calculated the data and tried to come to a convincing conclusion. We considered our results more reliable, for all the studies included were of high quality and we removed the duplicated data provided by Li and Huang et al. [75, 76]. As described above, the cumulative result provided a more powerful evidence and we intended to draw a conclusion that MPO-463G > A polymorphism may not be a good predictor of lung cancer both in Caucasians or Asians.

We first conducted a digestive system cancer group in meta-analysis and found significant results in allele frequency and dominant models. When it was divided into digestive tract and digestive gland, results indicated that digestive gland was more closely linked with decreasing cancer risk. Similarly, Samart et al. [43] even found that A allele and G/A or A/A separately reduced the risk of hepatoblastoma of 50 and 56% in Caucasians. Our analysis demonstrated that A allele and AG/AA had a 0.71-fold cancer risk and 0.67-fold cancer risk separately.

As for breast cancer, though positive result had been published before [11], whether it had a relationship with high level of MPO-G463 > A polymorphism stayed confusing. Our result for breast cancer was in line with the result by Chu et al. [9], suggesting a non-significant association. In addition, giving to the fact the level of MPO-containing neutrophils were high in breast tissue with or without cancer [77, 78], we tend to believe that MPO-463G > A polymorphism could not predict breast cancer well. A similar situation was seen in the blood system group, and the current study suggested no significant association between it and MPO-463G > A polymorphism.

Regarding the source of controls, the results should also be concerned. Only in the hospital population, significant results could be observed. This might reflect influence of health status, gene-gene or gene–environment interactions. Well-matched controls should be included in the future studies. Ethnicity is often one of the sources of heterogeneity. Stratifying the ethnicity, strength was observed in Caucasians and Asians under almost all the genetic models instead of the mix ethnicity group. Meanwhile, no significant association between MPO-463G > A polymorphism and digestive system cancer for Caucasians or lung cancer for Asians were found in any genetic model. Accordingly, we suggest the main source of heterogeneity is from the ethnicities, source of controls, and different cancer itself. And it may have something to do with age, sex, sample size, ethnic background, gene-gene interaction, environment background, and lifestyle. So it is meaningful to discuss this association more detailed. For example, analyses striated by age, sex, and accumulation of sample size are considerable.

Limited data of digestive system cancer and blood system cancer are one of the deficiencies of our meta-analysis. In addition, gene-gene interactions, environment background, and lifestyle were not well addressed in the meta-analysis for the lack of data. Taking it into consideration, more studies aimed to discuss the relationship of MPO-463G > A polymorphism and digestive system cancer, blood system cancer or the gene-gene or gene–environment interactions should be conducted to give a more deeper knowledge of this association. Besides, the lack of original information about age also limited our more in-depth research. We hope future researches can provide detailed information about age and then the stratified analysis by age could be done.

## Conclusion

Overall, in cumulative analysis, the stable trend indicated that evidence was sufficient to show the association between MPO-463G > A polymorphism and cancer risk. MPO-463G > A polymorphism might have an effect in reducing the risk of digestive system cancer, but might not be a good predictor of lung cancer, breast cancer, and blood system cancers. It was significantly associated with cancer risk both in Caucasians and Asians. But no significant association between MPO-463G > A polymorphism and Caucasians was found in any genetic model for digestive system cancer and no significant association between MPO-463G > A polymorphism and Asians was found for lung cancer. More studies exploring the association between MPO-463G > A polymorphism and digestive and blood system cancer are needed in the future.
